# Scatterers of Non-Electric-Dipole Radiation

**DOI:** 10.3390/nano15201584

**Published:** 2025-10-17

**Authors:** Yafei Li, Zhihui Liu, Shuanglong Cheng, Mansha Li, Jianchao Meng, Tao Jiang, Jiani Li, Zhuangzhuang Xu, Xike Qian, Meng Wang, Ze Li

**Affiliations:** 1 Key Laboratory of Semiconductor Photovoltaic Technology and Energy Materials of Inner Mongolia Autonomous Region, School of Physical Science and Technology, Inner Mongolia University, Hohhot 010021, China; 2 Research Center for Quantum Physics and Technologies, Inner Mongolia University, Hohhot 010021, China; 3 School of Science, Inner Mongolia University of Technology, Hohhot 010051, China

**Keywords:** electric dipole mode-free scatterer, anapole mode, nanodisk dimer

## Abstract

We theoretically demonstrate that nonmagnetic silicon nanodisk dimers, under plane-wave illumination, can achieve electric dipole mode-free by suppressing electric dipole responses at magnetic resonance frequencies through structural parameter tuning. This is enabled by the anapole mode, where destructive interference between Cartesian electric and toroidal dipole moments results in low spherical electric dipole scattering. Furthermore, the magnetic resonance responses in this nanostructure are tunable within the visible spectrum and compatible with current nanofabrication technology.

## 1. Introduction

Under the framework of classical electromagnetism, the magnetic field component of light is inherently several orders of magnitude weaker than its electric field counterpart. This fundamental intensity discrepancy has directly led to a long-standing imbalance in the research focus within the field of light-matter interactions, consigning interactions involving the magnetic field component of light to a state of prolonged neglect [1,2,3,4,5]. However, the development of functional optical metamaterials necessitates the realization of robust and effective optical magnetism [6,7,8]. To address this bottleneck, researchers have turned their attention to plasmonic nanostructures: the plasmon-induced magnetic resonance (PIMR) mode in such structures originates from the collective oscillation of free electrons in plasmonic materials, and this mode enables targeted modulation of the magnetic field component of light [7,8,9,10,11,12,13,14,15,16,17,18]. Whether in magnetic-resonance “split-ring resonator (SRR)” models analogous to optical nanocircuits [12,13,19] or magnetic dipole (MD) modes supported in multi-nanoparticle resonators [8,9,11,15,16,17], the generation of artificial magnetic moments relies on electric displacement vector loops associated with plasmonic electric multipolar modes [8,11,14]. These systems involve Fano coupling between weak MD modes and dominant electric multipoles, leading to magnetic-based Fano dips in scattering spectra [8,9,11]. Additionally, PIMR characteristics are highly sensitive to nanogap precision, requiring complex fabrication techniques such as molecular layer deposition to achieve the necessary dimensional control [8,11]. The significant intrinsic ohmic losses in metallic materials degrade the quality factor of magnetic resonances, severely limiting their practical applications [20]. Furthermore, the spectral overlap between MD and electric multipolar modes hinders the realization of pure magnetic responses, posing a critical challenge for enhancing magnetic interactions [2].

Recent investigations into Mie resonant dielectric nanostructures have demonstrated their remarkable ability to engineer magnetic resonance scattering at optical frequencies, simultaneously offering solutions to these challenges [21,22,23,24,25,26]. Although MD and magnetic quadrupole (MQ) modes in single silicon nanoparticles are clearly distinguishable in scattering spectra [21,27], they inherently suffer from spectral overlap with electric dipole (ED) modes, a fundamental limitation that precludes the realization of pure magnetic responses in such structures [2]. Thus, suppression of the ED response is essential for achieving electric dipole mode-free. Anapole states, associated with destructive interference between Cartesian electric and toroidal dipole moments, recently proposed in theoretical and experimental studies, offer a viable solution [28,29,30]. By enabling scattering cancelation between these two dipole moments, complete suppression of spherical ED scattering is achieved; spectral overlap with magnetic resonance modes then facilitates the realization of an electric dipole mode-free [31,32,33,34,35]. Although specific ideal MD resonances in gold core silicon shell nanospheres satisfy these conditions [2], the untuned magnetic resonance wavelengths in the near-infrared region and the stringent structural requirements imposed by cutting-edge photolithographic fabrication processes severely constrain the practical application of this method.

To address this issue, we demonstrate that silicon nanodisk dimers can serve as scatterers that enable electric dipole mode-free within the visible spectral range. This is accomplished by spectrally overlapping MD resonance, MQ resonance, and the nonradiative anapole mode [31,36], which enables the MD and MQ to dominate and suppresses the ED response at the same frequency. This designed disk-shaped structure offers unique advantages for practical applications. It is fully compatible with current nanofabrication technologies and establishes a new paradigm for light-matter interactions in dielectric nanostructures. It provides fundamental insights into magneto-optical phenomena and offers a practical platform for the development of functional optical metamaterials.

## 2. Results and Discussion

We used the Lumerical FDTD (Finite-Difference Time-Domain) simulation software (Version: 8.33.4048) to characterize the far-field optical magnetic responses of silicon nanodisk and nanodisk dimer structures. The geometric model used in these simulations is identical to the schematic structures presented in the following figures, and the dielectric constant of silicon was obtained by interpolating Palik’s experimental data. A linearly polarized total-field scattered-field (TFSF) source was employed as the excitation source, with the plane wave incident on the nanoparticles along the horizontal direction (x-axis), and the polarization direction could be set arbitrarily. In the scattered-field region, we configured a closed monitor array consisting of six discrete Fourier transform (DFT) monitors to measure the total scattering cross-section. To eliminate spurious reflections at the boundaries of the simulation domain, perfectly matched layer (PML) boundary conditions were adopted. The simulation runtime was set to 1000 femtoseconds (fs) to ensure sufficient convergence of the results. For optimizing computational efficiency, a uniform Yee mesh was used, with a mesh cell size of 2 nm × 2 nm × 2 nm. During the multipole decomposition of the dimer, a 3D DFT monitor was paired with a co-located 3D index monitor of the same dimensions to capture the electric field distributions required for mode analysis.

**Figure 1:**
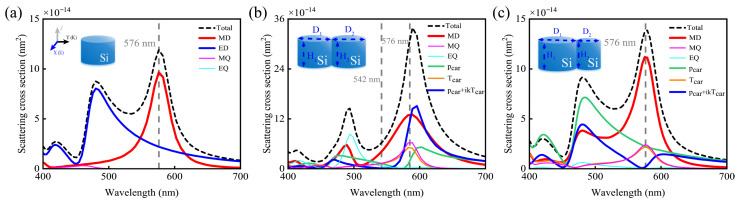
(**a**) Simulated scattering spectra of a single silicon nanodisk with diameter 140 nm and height 80 nm in air, along with the contributions of spherical ED and MD to the total scattering, where *δ*_Mag_ = 82%. (**b**) Simulated scattering spectra of a symmetric silicon nanodisk dimer with D_1_ = D_2_ = 140 nm, H_1_ = H_2_ = 80 nm, and gap size = 0 nm, together with the contributions of different multipolar moments to the total scattering, where *δ*_Mag_ = 88%. (**c**) Simulated scattering spectra of an asymmetric silicon nanodisk dimer with D_1_ = 140 nm, D_2_ = 40 nm, H_1_ = H_2_ = 80 nm, and gap size = 0 nm, along with the contributions of different multipolar moments to the total scattering, where *δ*_Mag_ = 98%.

In Figure 1a, we present the scattering spectrum of a single silicon nanodisk with a diameter of 140 nm and height of 80 nm, which exhibits two distinct peaks at 480 nm and 576 nm. To quantify the far-field scattering contributions of individual electromagnetic modes at these peak positions, we employ an exact spherical multipole expansion method for quantitative characterization. The different moments in the multipole expansion are computed from the calculated distribution of polarization current $J(r)=-i\omega P(r)=-i\omega \varepsilon_{0}(\varepsilon_{r}-1)E(r)$, here, **P** is the polarization vector, ω is the angular frequency, $\varepsilon_{r}$ represents the relative permittivity of nanoparticle, and E(r) denotes the total electric field induced inside the particle. The multipole moments of the ED, MD, electric quadrupole (EQ), and MQ can be derived as follows [11,23,37,38,39]:

(1) $${ED:p}_{\alpha }=-\frac{1}{i\omega }\left\{\int d^{3}rJ_{\alpha }^{\omega }j_{0}\left(kr\right)+\frac{k^{2}}{2}\int d^{3}r\left[3\left(r\cdot J_{\omega }\right)r_{\alpha }-r^{2}J_{\alpha }^{\omega }\right]\frac{j_{2\left(kr\right)}}{{\left(kr\right)}^{2}}\right\},$$

(2) $${MD:m}_{\alpha }=\frac{3}{2}\int d^{3}r{\left(r\times J_{\omega }\right)}_{\alpha }\frac{j_{1}\left(kr\right)}{kr},$$

(3) $$Q_{\alpha \beta }^{e}=-\frac{3}{i\omega }\left\{\begin{array}{c} \int d^{3}r\left[3\left(r_{\beta }J_{\alpha }^{\omega }+r_{\alpha }J_{\beta }^{\omega }\right)-2\left(r\cdot J_{\omega }\right)\delta_{\alpha \beta }\right]\frac{j_{1}(kr)}{kr} \end{array}\right.\;\;\left.+2k^{2}\int d^{3}r\left[5r_{\alpha }r_{\beta }\left(r\cdot J_{\omega }\right)-\left(r_{\alpha }J_{\beta }+r_{\alpha }J_{\beta }\right)r^{2}-r^{2}\left(r\cdot J_{\omega }\right)\delta_{\alpha \beta }\right]\frac{j_{3\left(kr\right)}}{{\left(kr\right)}^{3}}\right\},$$

(4) $${MQ:Q}_{\alpha \beta }^{m}=15\int d^{3}r\left\{r_{\alpha }{\left(r\times J_{\omega }\right)}_{\beta }+r_{\beta }{\left(r\times J_{\omega }\right)}_{\alpha }\right\}\frac{j_{2}\left(kr\right)}{{\left(kr\right)}^{2}},$$

k is wavenumber, $\alpha ,\beta =x,y,z{.p}_{\alpha }$, $m_{\alpha }$, $Q_{\alpha \beta }^{e}$, and $Q_{\alpha \beta }^{m}$ are the spherical multipole moments expressed in the Cartesian Coordinates where $j_{0,1,2}$ is spherical Bessel function of the zeroth, first, and second order. Using the multipole moments, it is easy to obtain the total scattering cross-section, i.e., the sum of the contributions from different multipole moments, as [37]:

(5) $$\begin{array}{l} C_{sca}^{total}=C_{sca}^{p}+C_{sca}^{m}+C_{sca}^{Q^{e}}+C_{sca}^{Q^{m}}\\ \;=\frac{k^{4}}{6\pi \varepsilon_{0}^{2}{\left|E_{0}\right|}^{2}}\left[\sum_{\alpha }\left({\left|p_{\alpha }\right|}^{2}+\frac{{\left|m_{\alpha }\right|}^{2}}{c}\right)+\frac{1}{120}\sum_{\alpha \beta }\left({\left|kQ_{\alpha \beta }^{e}\right|}^{2}+{\left|\frac{kQ_{\alpha \beta }^{m}}{c}\right|}^{2}\right)\right] \end{array}$$

where |**E**_0_| is the electric-field amplitude of the incident light, k is the wave number, and c is the speed of light.

As depicted in Figure 1a, the MD mode dominates at the 576 nm spectral peak, though notable spectral overlap with the ED mode is observed. Consequently, a single silicon nanodisk does not constitute an electric dipole mode-free scatterer. It is well established that the anapole mode—arising from destructive interference between Cartesian electric ($p_{Car}$) and toroidal dipole ($T_{Car}$) moments—results in low spherical ED scattering. By achieving spectral overlap between MD resonance and the anapole mode, electric dipole mode-free can be realized. Given that the excitation of an anapole mode necessitates a toroidal electric dipole, which comprises two oppositely oriented MD [35,39], we design an asymmetric silicon nanodisk dimer consisting of unequal-sized nanodisk to facilitate the excitation of the electric toroidal dipole ($T_{Car}$) mode.

To validate the effective excitation of the toroidal dipole and anapole mode in the dimer system, we calculate the scattering efficiencies of the Cartesian electric dipole ($p_{Car}$) and $T_{Car}$ in the Cartesian coordinate system. $T_{Car}$ moments emerge as higher-order terms in the expansion of ED moments: $ED=p_{Car}+ikT_{Car}$, specifically derived by applying the small-argument approximation to spherical Bessel functions: $j_{0}\left(k_{0}r\right)\approx 1-(k_{0}r{)}^{2}/6$, $j_{1}\left(k_{0}r\right)\approx k_{0}r/3$, $j_{2}\left(k_{0}r\right)\approx (k_{0}r{)}^{2}/15$. So, the multipole moments of $p_{Car}$ and $T_{Car}$ can be expressed as follows [31,37,38,39]:

(6) $$p_{Car}=\frac{1}{-i\omega }\int d^{3}rJ\left(r\right)$$

(7) $$T_{Car}=\frac{1}{10c}\int d^{3}r\left[\left(r\cdot J\left(r\right)\right)r-2r^{2}J\left(r\right)\right]$$

Moreover, the scattering cross-section in vacuum can be approximately calculated using Cartesian moments as [39]:

(8) $$\sigma_{sca}\approx \frac{k^{4}}{6\pi \varepsilon_{0}^{2}|E_{0}{|}^{2}}|p_{Car}+ikT_{Car}{|}^{2}+\frac{k^{4}\mu_{0}}{6\pi \varepsilon_{0}|E_{0}{|}^{2}}|m_{\alpha }{|}^{2}\;\;+\frac{k^{6}}{720\pi \varepsilon_{0}^{2}|E_{0}{|}^{2}}\sum |{\hat{Q}}_{\alpha \beta }^{c}{|}^{2}+\frac{k^{6}\mu_{0}}{80\pi \varepsilon_{0}|E_{0}{|}^{2}}\sum |{\hat{Q}}_{\alpha \beta }^{m}{|}^{2}$$

When irradiating the structure in the inset of Figure 1b (with diameters D_1_ = D_2_ = 140 nm, heights H_1_ = H_2_ = 80 nm, and gap size = 0 nm) using the same incident light, Figure 1b shows that the $p_{Car}$ mode and $T_{Car}$ mode undergo destructive interference at a wavelength of 542 nm. This interference leads to the complete suppression of the spherical ED response at this wavelength, while the MD mode remains dominant at 576 nm, accompanied by a resonance peak of the MQ mode. Subsequently, we investigated an asymmetric silicon nanodisk dimer with the diameter of D_2_ reduced to 40 nm, as depicted in Figure 1c. Here, the spectral intersection of the $p_{Car}$ and $T_{Car}$ modes redshifts to 576 nm, while the resonance peaks of the **MD** and **MQ** modes remain unshifted. This results in a precise alignment between the low ED scattering dip and magnetic resonances at 576 nm, enabling electric dipole mode-free. Another way to verify this conclusion is to quantitatively calculate the magnetic mode purity using the following formula,

(9) $$\delta_{Mag}=\delta_{MD}{+\delta }_{MQ}=\frac{C_{MD}+C_{MQ}}{C_{Sum}}\;=\frac{C_{MD}+C_{MQ}}{C_{MD}+C_{ED}+C_{MQ}+C_{EQ}+\ldots }\;\;\approx \frac{C_{MD}+C_{MQ}}{C_{MD}+C_{ED}+C_{MQ}+C_{EQ}}$$

where *δ*_Mag_ is the mode-purity factor of the magnetic resonance. C_MD_, C_ED_, C_MQ_ and C_EQ_ denote the scattering cross-section intensity of the **MD**, **ED**, **MQ** and **EQ** modes, respectively, all decomposed from the total scattering cross-section by performing a multipole expansion of C_Sum_ [22]. And it is found that the dimer structure with symmetry breaking has a magnetic mode purity of 98%.

Compared with a single silicon nanodisk, the total scattering intensity of the dimer system is moderately enhanced, demonstrating that dimer structures can more efficiently emulate high-purity magnetic resonance scatterers than isolated nanoparticles.

**Figure 2:**
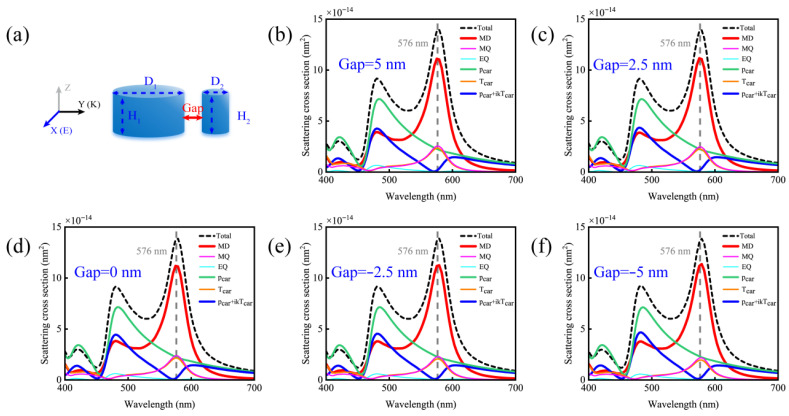
(**a**) Schematic diagram of the simulated scattering cross-section and multipole mode decomposition of a silicon disk dimer with D_1_ = 140 nm, D_2_ = 40 nm, and H_1_ = H_2_ = 80 nm as a function of the gap size; (**b**) with a gap of 5 nm; (**c**) with a gap of 2.5 nm; (**d**) with a gap of 0 nm, corresponds to the two nanodisks being in perfect contact; (**e**) with a gap of −2.5 nm; (**f**) with a gap of −5 nm. Negative gap values indicate an overlap between the structures.

We further investigated the influence of structural parameters of silicon nanodisk dimers on electric dipole mode-free scatterer. First, we simulated the scattering cross-sections and multipolar mode decompositions of dimers with D_1_ = 140 nm, D_2_ = 40 nm, and H_1_ = H_2_ = 80 nm at different gap sizes of 5 nm, 2.5 nm, 0 nm, −2.5 nm, and −5 nm, as shown in Figure 2. Here, a gap of 0 nm corresponds to the two nanodisks being in perfect contact, and negative gap values indicate an overlap between the structures. The dashed line in the figure denotes the wavelength of the electric dipole mode-free (576 nm). We find that the anapole mode closely aligns with the 576 nm wavelength of electric dipole mode-free across all gap sizes examined. This alignment indicates that the high-purity magnetic response is stably maintained at this wavelength, confirming that electric dipole mode-free scattering in silicon nanodisk dimers is independent of the gap size.

**Figure 3:**
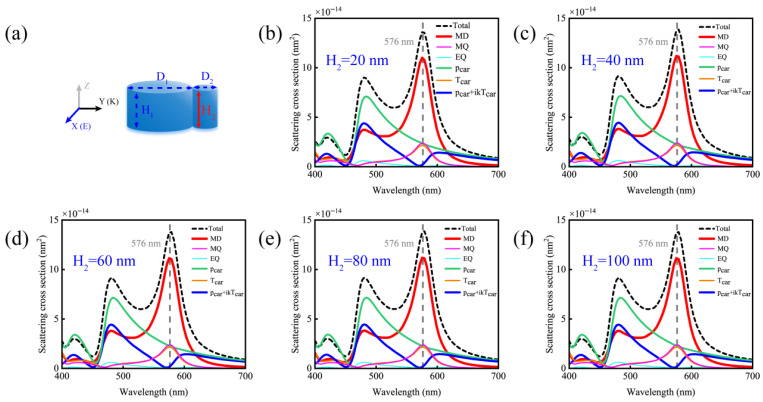
(**a**) Schematic diagram of the simulated contributions of multipole moments of a silicon disk dimer with D_1_ = 140 nm, D_2_ = 40 nm and H_1_ = 80 nm under different heights H_2_ of the smaller disk. (**b**) H_2_ = 20 nm; (**c**) H_2_ = 40 nm; (**d**) H_2_ = 60 nm; (**e**) H_2_ = 80 nm; (**f**) H_2_ = 100 nm.

To gain a deeper understanding of the physical mechanisms behind electric dipole mode-free in silicon nanodisk dimers, we simulated the contributions of different multipolar moments. When adjusting the height of the smaller nanodisk (as shown in Figure 3), the spectrum of the anapole mode remains consistently aligned with the electric dipole mode-free wavelength of 576 nm across all H_2_ values. This result indicates that the perfect scattering property of electric dipole mode-free is insensitive to the height of the smaller nanodisk, and the dimer can still maintain stable electric dipole mode-free performance within this parameter variation range.

**Figure 4:**
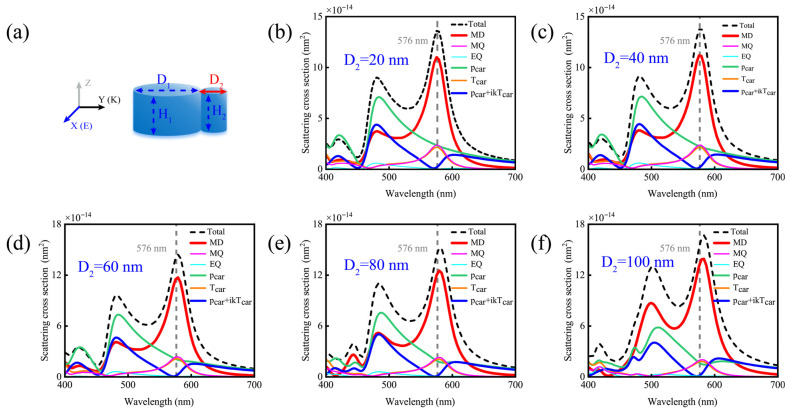
(**a**) Schematic diagram of the simulated multipole moment contributions of the silicon nanodisk dimer with D_1_ = 140 nm and H_1_ = H_2_ = 80 nm as the diameter D_2_ of the smaller disk varies. (**b**) D_2_ = 20 nm; (**c**) D_2_ = 40 nm; (**d**) D_2_ = 60 nm; (**e**) D_2_ = 80 nm; (**f**) D_2_ = 100 nm.

When adjusting the diameter of the smaller nanodisk, as shown in Figure 4. The MD and MQ mode peaks remain invariant at 576 nm across all cases. Although subtle variations in the anapole mode are observable in Figure 4f, its zero-response condition is tightly maintained at the 576 nm wavelength. These minor deviations arise exclusively from alterations in the spectral crossover point induced by changes in the relative intensities of $p_{Car}$ and $T_{Car}$ responses. Collectively, while the smaller nanodisk aids in anapole mode excitation, its geometric parameters exert no influence on the wavelength of the MD resonance peak. It is important to note that the results in Figure 2, Figure 3 and Figure 4 are very similar, demonstrating that the proposed electric dipole mode-free scatterer exhibits near-ideal stability against minor fabrication errors, such as variations in gap or the smaller nanodisk size. This robustness highlights the structure’s strong promise for practical applications.

**Figure 5:**
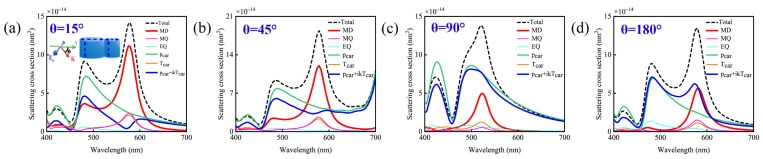
Schematic diagram of the simulated scattering cross-section and multipole mode decomposition of a silicon disk dimer with D_1_ = 140 nm, D_2_ = 40 nm, H_1_ = H_2_ = 80 nm and gap size = 0 nm as a function of the incident light angle. (**a**) θ = 15°; (**b**) θ = 45°; (**c**) θ = 90°; (**d**) θ = 180°.

To elucidate the physical mechanisms underlying the anapole mode wavelength shifts induced by changes in response intensities, we investigate the dimer structure under varying incident angles. As shown in the inset of Figure 5a, we simulate the scattering cross-sections and multipolar mode decompositions of a silicon nanodisk dimer with D_1_ = 140 nm, D_2_ = 40 nm, H_1_ = H_2_ = 80 nm, and gap size = 0 nm, defining the angle between the incident direction and the horizontal Y-axis as θ. For comparison with previous data (all calculated at θ = 0°), we analyze scattering and mode decompositions at θ = 15°, 45°, 90°, and 180°. Our results indicate that within the 0–90° incident angle range, the relative intensities of the $p_{Car}$ and $T_{Car}$ modes evolve with increasing angle, weakening the destructive interference between them and giving rise to a non-zero spherical ED response. Notably, for θ ≥ 90°, illumination by reflected light induces complete decoupling between $T_{Car}$ and $p_{Car}$, abolishing the electric dipole mode-free scattering behavior in the dimer structure. Therefore, although the system exhibits notable resilience to common fabrication imperfections (e.g., slight geometric variations), accurate control of the incident angle remains essential for optimal magnetic dipole resonance performance.

Through systematic investigations of the gap size, parameters of the smaller nanodisk, and incident angles, we first find that variations in the gap size have no influence on the excitation of the anapole mode, which lays a foundation for the structural robustness. Meanwhile, the smaller nanodisk facilitates the generation of MQ and **T**_Car_ modes without altering the resonance wavelength, thereby further ensuring the robust performance of the dimer structure. We deduce that the resonance wavelength of the MD in the dimer is predominantly regulated by the parameters of the larger nanodisk and the diameter matching-relationship between the larger and smaller nanodisks influences the relative intensities of the **p**_Car_ and **T**_Car_ responses, thereby enabling the fine-tuning of the wavelength position of the anapole mode. Additionally, direct illumination of the smaller disk prevents the excitation of the anapole mode, as the destructive interplay between $p_{Car}$ and $T_{Car}$ required for its formation is disrupted under such conditions.

**Figure 6:**
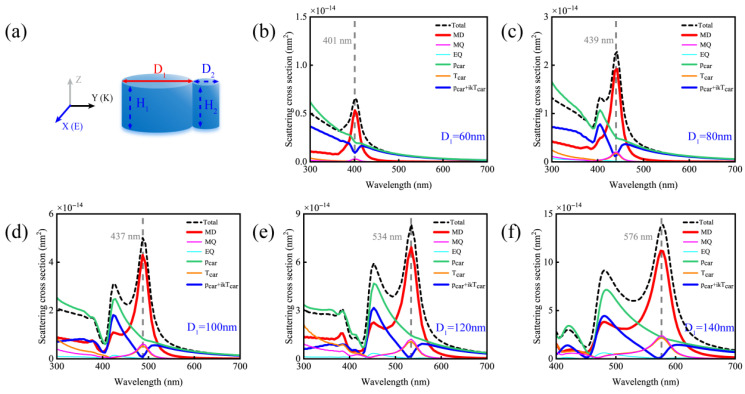
(**a**) Schematic diagram of the simulated changes in the contributions of different multipole moments of a silicon disk dimer with D_2_ = 40 nm and H_1_ = H_2_ = 80 nm as the diameter D_1_ of the larger disk varies. (**b**) D_1_ = 60 nm; (**c**) D_1_ = 80 nm; (**d**) D_1_ = 100 nm; (**e**) D_1_ = 120 nm; (**f**) D_1_ = 140 nm.

To further validate the theoretical inferences above, we analyze the contributions of different multipolar moments in dimers under varying parameters of the larger nanodisk. As shown in Figure 6, altering the diameter D_1_ of the larger disk induces shifts in the MD resonance peak and modifies the relative response intensities of the $p_{Car}$ and $T_{Car}$ modes. Because the MD resonance arises when the silicon nanoparticle size satisfies the condition: $\lambda_{0}\approx n_{Si}\cdot D_{Si}$ [21,31], where D_Si_ is the diameter of the silicon nanoparticle, $n_{Si}$ is the refractive index of silicon, and *λ*_0_ is the free-space wavelength. Specifically, for D_1_ = 140 nm and D_2_ = 40 nm, the spectral intersection of $p_{Car}$ and $T_{Car}$ responses gives rise to perfect destructive interference ($p_{Car}+ikT_{Car}=0$), directly validating the tuning effect of the diameter matching between the larger and smaller disks on the anapole mode position.

**Figure 7:**
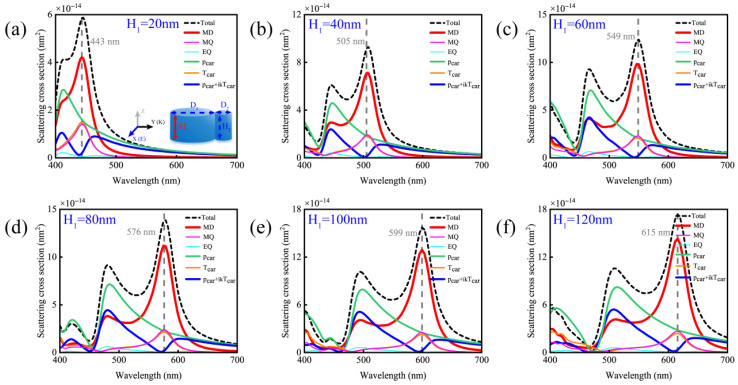
Schematic diagram of the simulated contributions of multipole moments of a silicon disk dimer with D_1_ = 140 nm, D_2_ = 40 nm and H_2_ = 80 nm under different heights H_1_ of the larger disk. (**a**) H_1_ = 20 nm; (**b**) H_1_ = 40 nm; (**c**) H_1_ = 60 nm; (**d**) H_1_ = 80 nm; (**e**) H_1_ = 100 nm; (**f**) H_1_ = 120 nm.

Finally, we investigate the contributions of different multipolar moments under variations in the larger nanodisk height, as shown in Figure 7. For a silicon nanodisk dimer with D_1_ = 140 nm and D_2_ = 40 nm, we find that perfect magnetic resonance scattering can be achieved across the entire visible spectral range by adjusting H_1_. Thus, once the diameter matching between D_1_ and D_2_ for perfect magnetic resonance scattering is established, tuning within the visible spectrum becomes feasible via H_1_ modulation. This provides a more convenient approach for implementing magnetic resonance scattering in silicon nanodisk dimers, enhancing their practical applicability.

**Figure 8:**
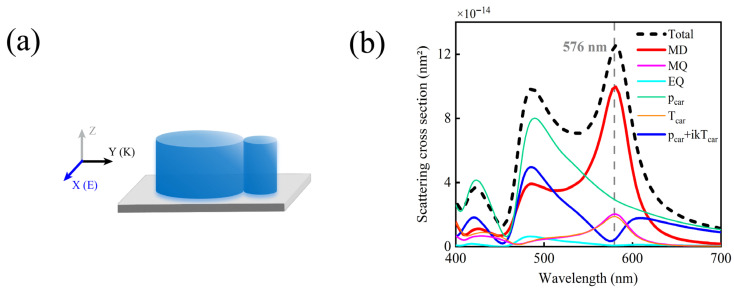
(**a**) Schematic diagram of the simulation for a silicon disk dimer (with D_1_ = 140 nm, D_2_ = 40 nm, and H_1_ = H_2_ = 80 nm) placed on a SiO_2_ substrate. (**b**) Simulated scattering spectrum of the silicon nanodisk dimer on the SiO_2_ substrate, along with the contributions of different multipole moments to the total scattering, where δ_Mag_ = 96%.

In practical experiments, silicon nanoparticles typically need to be placed on dielectric substrates, a step that is unavoidable in the fabrication process. As shown in Figure 8b, after introducing a silicon dioxide (SiO_2_) substrate, the low electric dipole scattering phenomenon induced by the anapole mode remains clearly observable at the **MD** resonance wavelength, with *δ*_Mag_ as high as 96%, which fully confirms the structural robustness of the anapole mode in resisting substrate-induced electromagnetic interactions. Furthermore, the performance can be further optimized through substrate etching technology, which enables the achievement of magnetic scattering with extremely high purity. This is of great significance for advancing the practical application of dielectric nanostructures in magnetic resonance-based nanophotonic devices.

## 3. Conclusions

In summary, through numerical simulations, we have verified that a silicon nanodisk dimer under plane-wave illumination can achieve electric dipole mode-free scattering in the far-field, where the MD and MQ overlap. This occurs due to the destructive interference between the $p_{Car}$ and $T_{Car}$ modes, leading to the formation of a non-radiative anapole mode. Moreover, we have found that the magnetic resonance wavelength of this structure is tunable within the visible light range. We have provided two tuning methods, and adjusting the height of the larger nanodisk offers a more convenient and rapid tuning approach. Our research results provide an ideal platform that is more conducive to practical applications for studying magneto-optical-matter interactions in non-magnetic materials.

## Data Availability

The original contributions presented in this study are included in the article. Further inquiries can be directed to the corresponding authors.
